# Supplementary material for: Polyketide synthase gene clusters in the ericoid mycorrhizal fungus Oidiodendron maius: genome-wide prediction, expression profiling, and functional characterization of knockout mutants
Source: Front Plant Sci. 2026 Sep 16;17:1924430. doi: 10.3389/fpls.2026.1924430 (PMC13623903; doi:10.3389/fpls.2026.1924430)
Supplement: Supplementary file 1 [file SupplementaryFile1.zip › Supplementary Materials/Supplementary Folder 1.zip!/Oidma1_AssemblyScaffolds_Repeatmasked/knownclusterblast/cluster24/jgi.p_Oidma1_125828_mibig_hits.html]

| MIBiG Protein | Description | MIBiG Cluster | MiBiG Product | % ID | % Coverage | BLAST Score | E-value |
| --- | --- | --- | --- | --- | --- | --- | --- |
| AGI62225.1 | AbeX1-like\_monooxygenase | BGC0001335 | Borregomycin\_biosynthetic\_gene\_cluster | 34 | 103.001876173 | 263 | 4.9e-70 |
| AEF32090.1 | monooxygenase | BGC0001333 | BE-54017\_biosynthetic\_gene\_cluster | 35 | 99.6247654784 | 255 | 1.8e-67 |
| AEO12714.1 | flavin\_monooxygenase | BGC0001334 | Cladoniamide\_biosynthetic\_gene\_cluster | 33 | 100.750469043 | 254 | 2.3e-67 |
| BAQ25478.1 | monooxygenase | BGC0001288 | Maklamicin\_biosynthetic\_gene\_cluster | 34 | 104.315196998 | 248 | 2.1e-65 |
| AKD43496.1 | Monooxygenase | BGC0001409 | Dutomycin\_biosynthetic\_gene\_cluster | 32 | 97.5609756098 | 243 | 6.9e-64 |
| ABI22123.1 | monooxygenase | BGC0000422 | Saframycin\_A\_biosynthetic\_gene\_cluster | 33 | 98.8742964353 | 237 | 4.9e-62 |
| AGD80608.1 | monooxygenase | BGC0000394 | Naphthyridinomycin\_biosynthetic\_gene\_cluster | 34 | 100.0 | 237 | 4.9e-62 |
| AEI98648.1 | CtcE | BGC0000209 | Chlortetracycline\_biosynthetic\_gene\_cluster | 33 | 97.373358349 | 231 | 2.7e-60 |
| CBW45655.1 | putative\_oxygenase | BGC0000952 | Pristinamycin\_biosynthetic\_gene\_cluster | 32 | 103.939962477 | 229 | 7.9e-60 |
| AGZ15446.1 | putative\_polyketide\_oxygenase/hydroxylase | BGC0001036 | Polyoxypeptin\_biosynthetic\_gene\_cluster | 33 | 98.1238273921 | 225 | 1.5e-58 |
| CAA07757.1 | oxygenase | BGC0000246 | Mithramycin\_biosynthetic\_gene\_cluster | 32 | 99.4371482176 | 221 | 2.8e-57 |
| CAK50777.1 | oxygenase | BGC0000247 | Mithramycin\_biosynthetic\_gene\_cluster | 32 | 99.4371482176 | 221 | 2.8e-57 |
| ACN64853.1 | PokO2 | BGC0001061 | Polyketomycin\_biosynthetic\_gene\_cluster | 32 | 97.373358349 | 219 | 1.1e-56 |
| ADE34483.1 | SsfO2 | BGC0000269 | SF2575\_biosynthetic\_gene\_cluster | 32 | 102.063789869 | 218 | 1.8e-56 |
| ADB02842.1 | AzicO4 | BGC0000202 | Azicemicin\_biosynthetic\_gene\_cluster | 38 | 68.2926829268 | 213 | 7.6e-55 |
| AAZ78335.1 | OxyL | BGC0000254 | Oxytetracycline\_biosynthetic\_gene\_cluster | 33 | 101.125703565 | 210 | 5e-54 |
| AAZ78342.1 | OxyS | BGC0000254 | Oxytetracycline\_biosynthetic\_gene\_cluster | 32 | 103.189493433 | 205 | 1.6e-52 |
| CAG14971.1 | oxygenase | BGC0000253 | Oviedomycin\_biosynthetic\_gene\_cluster | 36 | 64.1651031895 | 205 | 2.1e-52 |
| AKQ20687.1 | monooxygenase | BGC0001337 | Lazarimide\_biosynthetic\_gene\_cluster | 36 | 66.4165103189 | 205 | 2.1e-52 |
| ACY06308.1 | FAD-dependent\_monooxygenase | BGC0001042 | Sanglifehrin\_A\_biosynthetic\_gene\_cluster | 34 | 64.5403377111 | 194 | 2.8e-49 |
| CAE17536.1 | oxygenase | BGC0000210 | Chromomycin\_A3\_biosynthetic\_gene\_cluster | 30 | 100.562851782 | 193 | 4.8e-49 |
| AAK57522.1 | PgaE | BGC0000262 | Rabelomycin\_biosynthetic\_gene\_cluster | 36 | 65.6660412758 | 192 | 1.4e-48 |
| EHM27503.1 | monooxygenase\_FAD-binding | BGC0000235 | Kiamycin\_biosynthetic\_gene\_cluster | 36 | 64.1651031895 | 190 | 4.1e-48 |
| ABO15838.1 | FAD-dependent\_monooxygenase | BGC0000130 | Pyrrolomycin\_biosynthetic\_gene\_cluster | 35 | 65.1031894934 | 190 | 5.3e-48 |
| AEE65473.1 | oxygenase-like\_protein | BGC0000223 | Fluostatin\_biosynthetic\_gene\_cluster | 37 | 66.0412757974 | 189 | 1.2e-47 |
| AAM54089.1 | oxygenase | BGC0000020 | Ansamitocin\_biosynthetic\_gene\_cluster | 36 | 75.7973733583 | 188 | 1.5e-47 |
| CAH10119.1 | putative\_oxygenase | BGC0000268 | Sch47554\_/\_Sch47555\_biosynthetic\_gene\_cluster | 37 | 64.7279549719 | 188 | 2e-47 |
| ADB02837.1 | AzicO3 | BGC0000202 | Azicemicin\_biosynthetic\_gene\_cluster | 36 | 66.4165103189 | 188 | 2.6e-47 |
| CAG14963.1 | oxygenase | BGC0000253 | Oviedomycin\_biosynthetic\_gene\_cluster | 39 | 64.7279549719 | 188 | 2.6e-47 |
| ADI71441.1 | putative\_oxygenase | BGC0000203 | BE-7585A\_biosynthetic\_gene\_cluster | 37 | 64.7279549719 | 188 | 2.6e-47 |
| AAX57188.2 | auricin\_polyketide\_oxiygenase | BGC0000201 | Auricin\_biosynthetic\_gene\_cluster | 37 | 64.9155722326 | 186 | 5.9e-47 |
| CAA60567.1 | putative\_oxygenase | BGC0000276\_c4 | Urdamycin\_biosynthetic\_gene\_cluster | 37 | 64.7279549719 | 185 | 1.3e-46 |
| AGO50609.1 | oxidoreductase | BGC0000229 | Grincamycin\_biosynthetic\_gene\_cluster | 37 | 65.1031894934 | 185 | 1.3e-46 |
| BAV16997.1 | putative\_oxygenase | BGC0001384 | Saprolmycin\_biosynthetic\_gene\_cluster | 36 | 64.9155722326 | 185 | 1.7e-46 |
| AAM97364.1 | RubN | BGC0000266 | Rubromycin\_biosynthetic\_gene\_cluster | 34 | 64.5403377111 | 185 | 1.7e-46 |
| AAX57196.2 | auricin\_polyketide\_oxygenase-reductase | BGC0000201 | Auricin\_biosynthetic\_gene\_cluster | 29 | 97.9362101313 | 184 | 2.9e-46 |
| ADQ55479.1 | FAD-binding\_monooxygenase | BGC0000350 | ET-743\_biosynthetic\_gene\_cluster | 29 | 75.9849906191 | 183 | 5e-46 |
| AAD13534.1 | oxygenase\_homolog | BGC0000239 | Landomycin\_biosynthetic\_gene\_cluster | 36 | 63.9774859287 | 181 | 1.9e-45 |
| ctg1\_orf39 |  | BGC0001013 | Meridamycin\_biosynthetic\_gene\_cluster | 33 | 77.4859287054 | 181 | 3.2e-45 |
| AFU65887.1 | DacO3 | BGC0000216 | Dactylocycline\_biosynthetic\_gene\_cluster | 35 | 65.6660412758 | 180 | 4.2e-45 |
| CAG14970.1 | oxygenase | BGC0000253 | Oviedomycin\_biosynthetic\_gene\_cluster | 32 | 98.8742964353 | 180 | 4.2e-45 |
| BAJ52699.1 | putative\_FAD-binding\_monooxygenase | BGC0000222 | FD-594\_biosynthetic\_gene\_cluster | 32 | 82.9268292683 | 180 | 4.2e-45 |
| ACP19351.1 | SaqE | BGC0000267 | Saquayamycin\_Z\_/\_galtamycin\_B\_biosynthetic\_gene\_cluster | 36 | 64.7279549719 | 180 | 5.5e-45 |
| AAK06782.1 | putative\_oxygenase\_SimA7 | BGC0001072 | Simocyclinone\_biosynthetic\_gene\_cluster | 34 | 64.7279549719 | 179 | 9.4e-45 |
| AEE65484.1 | monooxygenase | BGC0000223 | Fluostatin\_biosynthetic\_gene\_cluster | 35 | 66.2288930582 | 179 | 9.4e-45 |
| CBH32814.1 | putative\_monooxygenase | BGC0000263 | Ravidomycin\_biosynthetic\_gene\_cluster | 30 | 100.562851782 | 177 | 3.6e-44 |
| ABX71099.1 | Lct16 | BGC0000238 | Lactonamycin\_biosynthetic\_gene\_cluster | 30 | 102.251407129 | 176 | 7.9e-44 |
| BAE93724.1 | putative\_FAD-dependent\_monooxygenase | BGC0000164 | Tetronomycin\_biosynthetic\_gene\_cluster | 34 | 64.9155722326 | 175 | 1.8e-43 |
| WP\_030269258.1 | monooxygenase | BGC0001308 | Simocyclinone\_D9\_/\_simocyclinone\_D10\_/\_simocyclinone\_D11 | 36 | 66.0412757974 | 174 | 3e-43 |
| AHZ61858.1 | Lom24 | BGC0000240 | Lomaiviticin\_biosynthetic\_gene\_cluster | 30 | 102.063789869 | 174 | 3.9e-43 |
| AAY28223.1 | HbmL | BGC0000074 | Herbimycin\_biosynthetic\_gene\_cluster | 33 | 85.3658536585 | 173 | 6.7e-43 |
| AAO65343.1 | oxygenase-like\_protein | BGC0000236 | Kinamycin\_biosynthetic\_gene\_cluster | 36 | 65.478424015 | 173 | 8.8e-43 |
| ADE34499.1 | SsfO1 | BGC0000269 | SF2575\_biosynthetic\_gene\_cluster | 33 | 66.6041275797 | 171 | 2.6e-42 |
| AEI98657.1 | CtcN | BGC0000209 | Chlortetracycline\_biosynthetic\_gene\_cluster | 33 | 70.1688555347 | 170 | 4.4e-42 |
| AAL15585.1 | Sim7 | BGC0000270 | Simocyclinone\_biosynthetic\_gene\_cluster | 33 | 65.8536585366 | 170 | 4.4e-42 |
| AAZ77692.1 | ChlE1 | BGC0000036 | Chlorothricin\_biosynthetic\_gene\_cluster | 33 | 69.04315197 | 170 | 7.4e-42 |
| BAJ07857.1 | putative\_oxygenase | BGC0000232 | Hatomarubigin\_biosynthetic\_gene\_cluster | 39 | 45.4033771107 | 169 | 9.7e-42 |
| CAO98853.1 | monooxygenase\_AufJ | BGC0000023 | Aurafuron\_biosynthetic\_gene\_cluster | 30 | 70.5440900563 | 169 | 1.3e-41 |
| ABP54647.1 | monooxygenase,\_FAD-binding | BGC0000241 | Lomaiviticin\_biosynthetic\_gene\_cluster | 36 | 65.478424015 | 168 | 1.7e-41 |
| AAO06914.1 | GdmL | BGC0000066 | Geldanamycin\_biosynthetic\_gene\_cluster | 33 | 84.990619137 | 168 | 2.2e-41 |
| CAK50794.2 | oxygenase | BGC0000247 | Mithramycin\_biosynthetic\_gene\_cluster | 33 | 66.4165103189 | 168 | 2.8e-41 |
| AAK57530.1 | PgaM | BGC0000262 | Rabelomycin\_biosynthetic\_gene\_cluster | 32 | 64.5403377111 | 166 | 1.1e-40 |
| ABB88524.1 | Baeyer-Villiger\_monooxygenase | BGC0000050 | E-837\_biosynthetic\_gene\_cluster | 29 | 97.5609756098 | 165 | 2.4e-40 |
| AAO65352.1 | oxygenase-like\_protein | BGC0000236 | Kinamycin\_biosynthetic\_gene\_cluster | 29 | 99.4371482176 | 164 | 3.1e-40 |
| AHE14649.1 | FAD-binding\_monooxygenase | BGC0001336 | Erdasporine\_biosynthetic\_gene\_cluster | 30 | 79.7373358349 | 164 | 3.1e-40 |
| CBH32083.1 | putative\_FAD-dependent\_monooxygenase | BGC0000211 | Chrysomycin\_biosynthetic\_gene\_cluster | 34 | 65.6660412758 | 163 | 6.9e-40 |
| AAD13541.1 | oxygenase\_homolog | BGC0000239 | Landomycin\_biosynthetic\_gene\_cluster | 32 | 63.7898686679 | 161 | 2e-39 |
| AAP69582.1 | putative\_FAD-dependent\_oxygenase | BGC0000226 | Gilvocarcin\_biosynthetic\_gene\_cluster | 35 | 65.478424015 | 161 | 2e-39 |
| AAZ77700.1 | ChlE3 | BGC0000036 | Chlorothricin\_biosynthetic\_gene\_cluster | 31 | 65.8536585366 | 160 | 5.9e-39 |
| ACR50781.1 | putative\_FAD-dependent\_oxygenase | BGC0000163 | Tetronasin\_biosynthetic\_gene\_cluster | 34 | 64.9155722326 | 156 | 8.5e-38 |
| ABC87527.1 | FAD-dependent\_monooxygenase | BGC0001011 | Meridamycin\_biosynthetic\_gene\_cluster | 32 | 71.8574108818 | 156 | 1.1e-37 |
| AFU65892.1 | DacO1 | BGC0000216 | Dactylocycline\_biosynthetic\_gene\_cluster | 31 | 71.1069418386 | 155 | 2.5e-37 |
| AAK06789.1 | putative\_oxygenase\_SimA8 | BGC0001072 | Simocyclinone\_biosynthetic\_gene\_cluster | 31 | 65.8536585366 | 150 | 4.7e-36 |
| ACB37744.1 | FAD-dependent\_oxygenase | BGC0000162 | Tetrocarcin\_A\_biosynthetic\_gene\_cluster | 31 | 65.478424015 | 148 | 1.8e-35 |
| AGO50615.1 | oxygenase-reductase | BGC0000229 | Grincamycin\_biosynthetic\_gene\_cluster | 31 | 65.2908067542 | 148 | 2.3e-35 |
| BAQ21949.1 | putative\_FAD-dependent\_oxidoreductase | BGC0001204 | Versipelostatin\_biosynthetic\_gene\_cluster | 28 | 69.04315197 | 141 | 3.7e-33 |
| AGI99493.1 | FAD-dependent\_oxidoreductase | BGC0001004 | Lobophorin\_biosynthetic\_gene\_cluster | 33 | 64.7279549719 | 140 | 4.8e-33 |
| ACB46484.1 | FAD-dependent\_oxidoreductase | BGC0000082 | Kijanimicin\_biosynthetic\_gene\_cluster | 39 | 41.6510318949 | 139 | 8.2e-33 |
| AGC09488.1 | LobA | BGC0001183 | Lobophorin\_biosynthetic\_gene\_cluster | 33 | 64.7279549719 | 138 | 1.8e-32 |
| AEE65478.1 | putative\_oxygenase | BGC0000223 | Fluostatin\_biosynthetic\_gene\_cluster | 32 | 65.6660412758 | 136 | 1.2e-31 |
| BAC10676.1 | putative\_FAD-monooxygenase | BGC0000821 | Rebeccamycin\_biosynthetic\_gene\_cluster | 28 | 105.065666041 | 135 | 1.6e-31 |
| CAC93716.1 | putative\_monooxygenase | BGC0000822 | Rebeccamycin\_biosynthetic\_gene\_cluster | 28 | 105.065666041 | 135 | 1.6e-31 |
| BAC15752.1 | RebC | BGC0000823 | Rebeccamycin\_biosynthetic\_gene\_cluster | 28 | 105.065666041 | 135 | 1.6e-31 |
| BAF47693.1 | monooxygenase | BGC0000825 | Staurosporine\_biosynthetic\_gene\_cluster | 31 | 70.3564727955 | 132 | 1.7e-30 |
| EWM63062.1 | FAD-binding\_monooxygenase | BGC0000679 | Diazepinomicin\_biosynthetic\_gene\_cluster | 30 | 78.2363977486 | 129 | 1.1e-29 |
| CAD19095.1 | cytochrome\_P450\_dependent\_monooxygenase | BGC0000153 | Stigmatellin\_biosynthetic\_gene\_cluster | 27 | 74.6716697936 | 125 | 1.6e-28 |
| AKG47162.1 | HysC | BGC0001223 | Hydroxysporine\_biosynthetic\_gene\_cluster | 30 | 66.2288930582 | 124 | 2.7e-28 |
| ABV98191.1 | monooxygenase\_FAD-binding | BGC0000827 | Staurosporine\_biosynthetic\_gene\_cluster | 29 | 65.2908067542 | 119 | 1.2e-26 |
| EFG04594.1 | Staurosporine\_biosynthesis\_monooxygenase\_StaC | BGC0000826 | Staurosporine\_biosynthetic\_gene\_cluster | 28 | 81.8011257036 | 114 | 3.7e-25 |
| AGI62219.1 | RebC-like\_monooxygenase | BGC0001335 | Borregomycin\_biosynthetic\_gene\_cluster | 28 | 77.861163227 | 113 | 1.1e-24 |
| ADE22302.1 | putative\_FAD-binding\_monooxygenase | BGC0000279 | Xantholipin\_biosynthetic\_gene\_cluster | 32 | 46.3414634146 | 111 | 3.1e-24 |
| ABC02791.1 | putative\_FAD-monooxygenase | BGC0000809 | AT2433\_biosynthetic\_gene\_cluster | 28 | 82.9268292683 | 109 | 1.6e-23 |
| AEO12712.1 | flavin\_monooxygenase | BGC0001334 | Cladoniamide\_biosynthetic\_gene\_cluster | 34 | 42.5891181989 | 102 | 1.1e-21 |
| CAM34363.1 | putative\_FAD-binding\_monooxygenase,\_PheA/TfdB\_family | BGC0000242 | Lysolipin\_biosynthetic\_gene\_cluster | 33 | 48.2176360225 | 99 | 1.6e-20 |
| BAB69162.1 | hydroxylase | BGC0000271 | Spore\_pigment\_biosynthetic\_gene\_cluster | 29 | 47.2795497186 | 93 | 8.8e-19 |
| CCH63734.1 | XiaH\_protein | BGC0000665 | Xiamycin\_biosynthetic\_gene\_cluster | 30 | 43.339587242 | 86 | 1.1e-16 |
| AFK78077.1 | XiaK | BGC0000666 | Xiamycin\_biosynthetic\_gene\_cluster | 30 | 43.339587242 | 86 | 1.1e-16 |
| AHL46732.1 | FAD\_dependent\_monooxygenase | BGC0001179 | Rishirilide\_B\_biosynthetic\_gene\_cluster | 30 | 48.7804878049 | 85 | 1.8e-16 |
| AFV30246.1 | FAD-binding\_monooxygenase | BGC0000075 | Hygrocin\_biosynthetic\_gene\_cluster | 34 | 41.0881801126 | 84 | 5.4e-16 |
